# Feasibility of Integrating a Standardized Psychosocial Follow‐Up After Childhood Cancer—An Interim Analysis of the LE‐Na Study

**DOI:** 10.1002/cam4.72148

**Published:** 2026-08-04

**Authors:** Madelaine Sleimann, Magdalena Balcerek, Gabriele Calaminus, Martina B. Schaefer, Rainer John, Judith Lohse, Sonja Schuster, Teresa Halbsguth, Alexander Puzik, Franziska Wolters, Jörg Faber, Irene von Luettichau, Sophia Hömberg, Sebastian Michaelis, Melanie Kaiser, Desiree Grabow, Inke König, Anke Neumann, Ann‐Kristin Kock‐Schoppenhauer, Celina Arendt, Thorsten Langer, Katja Baust, Judith Gebauer

**Affiliations:** ^1^ Department of Pediatrics University Hospital Schleswig‐Holstein Lübeck Germany; ^2^ Department of Pediatric Oncology and Hematology Charité–Universitätsmedizin Berlin, corporate Member of Freie Universität Berlin and Humboldt‐Universität zu Berlin Berlin Germany; ^3^ Department of Oncology, Hepatology and Pneumology University Hospital Leipzig and University Cancer Center Leipzig Leipzig Germany; ^4^ Clinic for Pediatric Hematology and Oncology, Center for Pediatric and Adolescent Medicine University Hospital Bonn Bonn Germany; ^5^ Clinic and Polyclinic for Pediatrics and Adolescent Medicine, Department of Pediatric Hematology and Oncology University Hospital Carl Gustav Carus, Technische Universität Dresden Dresden Germany; ^6^ Department of Pediatric Hematology and Oncology, Department of Pediatrics and Adolescent Medicine University Hospital Erlangen, Friedrich‐Alexander‐Universität Erlangen‐Nürnberg (FAU) Erlangen Germany; ^7^ Department of Medicine II, Hematology and Oncology, University Cancer Center (UCT) Frankfurt University Hospital Frankfurt, Goethe University Frankfurt Frankfurt am Main Germany; ^8^ Department of Pediatric Hematology, Oncology and Stem Cell Transplantation, Children's Hospital, Medical Center, Faculty of Medicine University of Freiburg Freiburg Germany; ^9^ Department of Hematology and Oncology With Section Pneumology, Hubertus Wald Tumor Center–University Cancer Center Hamburg University Medical Center Hamburg‐Eppendorf (UKE) Hamburg Germany; ^10^ Department of Pediatric Hematology/Oncology, Department of Pediatrics and Adolescent Medicine University Medical Center Mainz Mainz Germany; ^11^ Center for Pediatrics and Adolescent Medicine, a Collaboration Between Munich Clinic and TUM University Hospital Rechts der Isar, TUM School of Medicine and Health Munich Germany; ^12^ Department of Pediatric Hematology and Oncology Asklepios Children's Hospital Sankt Augustin Sankt Augustin Germany; ^13^ Department I of Pediatric Hematology and Oncology University Children's Hospital Tübingen Tübingen Germany; ^14^ Department of Epidemiology of Childhood Cancer (EpiKiK)/German Childhood Cancer Registry (DKKR), Institute for Medical Biostatistics, Epidemiology and Informatics (IMBEI) University Medical Center of the Johannes Gutenberg University Mainz Mainz Germany; ^15^ Institute of Medical Biometry and Statistics (IMBS), University of Luebeck, University Hospital Schleswig‐Holstein Lübeck Germany; ^16^ Section for Clinical Research IT (SKFIT) Institute of Medical Biometry and Statistics (IMBS), University of Luebeck and University Hospital Schleswig‐Holstein Lübeck Germany; ^17^ Department of Pediatric Hematology and Oncology, Department of Pediatrics and Adolescent Medicine University Hospital Schleswig‐Holstein Lübeck Germany; ^18^ Department of Hematology and Oncology University Hospital Schleswig‐Holstein Lübeck Germany

**Keywords:** childhood cancer survivors, late effects, long‐term follow‐up, mental health, psychosocial well‐being, quality of life

## Abstract

**Background:**

Many childhood and adolescent cancer survivors suffer from late effects that impair quality of life and life expectancy. However, only an estimated 5% of > 47,000 survivors in Germany receive adequate long‐term follow‐up (LTFU) care. The LE‐Na (Evaluation and implementation of multidisciplinary, standardized, guideline‐based long‐term follow‐up care for adult survivors of childhood cancer in Germany) study aims to address this care gap. Our interim analysis compares three recruitment and information pathways and provides information on the feasibility and acceptance of a guideline‐based psychosocial screening.

**Methods:**

Over the total study period of 5 years, 5000 survivors without previous LTFU care are systematically invited to specialized LTFU clinics in Germany: (1) through the German Childhood Cancer Registry (GCCR), (2) during transition from pediatric to adult care, and (3) via media presence. Longitudinal sociodemographic, medical, and psychosocial data are documented in an online database. Follow‐up intervals are based on therapy‐associated risk for late effects: annually (high risk), biannually (medium risk), or every 5 years (low risk).

**Results:**

By the time of our interim analysis, 300 survivors with completed data documentation had been enrolled: 121 (40.3%) were recruited via GCCR invitations, 155 (51.7%) through a structured transition, and 24 (8%) via media presence. Nearly half (131/300; 43.7%) were at high risk for late effects. Almost all (299/300, 99.7%) completed psychosocial screening. A Distress‐Thermometer score of ≥ 5 was found in half of the participants (146/299; 48.8%), indicating relevant psychosocial distress.

**Conclusion:**

Through the different recruitment methods, survivors with a high risk for late effects were particularly encouraged to accept the LTFU care offer. Integrating a psychosocial screening is feasible and confirms survivors' distress.

**Trial Registration:**

DRKS identifier: DRKS00033303

## 1 Background

Increasing long‐term survival rates of cancer diagnosed before the age of 18 years result in a constantly growing number of childhood cancer survivors (CCSs) worldwide [1]. In Germany, over 46,000 CCSs, who have survived their disease for 5 years or more, have been registered in the German Childhood Cancer Registry (GCCR) since 1980 [2]. The GCCR regularly carries out a questionnaire‐based follow‐up to track mortality, relapses, and subsequent neoplasms as well as maintain current contact details from this cohort as part of mandated duties [2, 3, 4, 5].

Even many years after a cure from the initial cancer, many CCS may experience cancer treatment‐related late effects resulting in increased morbidity and reduced health‐related quality of life [6, 7]. As these may present as complex chronic physical and mental health impairments, including fatigue [8, 9], CCS require risk‐adapted long‐term follow‐up (LTFU) care aiming at diagnosing and treating late effects in time. Despite increasing evidence that multidisciplinary life‐long LTFU reduces mortality and both physical and mental morbidity [6, 10, 11], optimal strategies to motivate CCS to engage in regular LTFU care remain unclear [12], particularly among those who have not yet been systematically informed about potential late effects and care recommendations. Ideally, a first approach avoids inducing fear and insecurity. While CCS usually receive continuous LTFU while treated in the pediatric oncology department, care likely is disrupted at transition time points, for example, when CCS enter adulthood. In Germany, many of the adult CCS do not receive regular LTFU care, especially concerning mental health issues.

Current guidelines recommend systematic screening for psychological disorders including psychological distress, mood disorders, anxiety, post‐traumatic stress disorder (PTSD) or related symptoms (PTSS) as well as fatigue [13] at every LTFU visit [9, 13]. Besides counseling on mental health issues, CCS with significant impairments should also receive adequate mental health interventions that consider their childhood or adolescent cancer history. Such screenings and targeted interventions are essential for promoting a healthy lifestyle and enhancing adherence to LTFU care, as conditions such as mood disorders may increase the likelihood of risky health behaviors or avoidance of medical care [9, 14]. In addition, social aspects need to be considered in LTFU care of CCS, as some are at risk for poor educational and occupational outcomes, resulting in early retirement, unemployment, or long‐term incapacity for work, sometimes influencing their ability to live independently from their parents or other care persons [15]. Vice versa, such limitations to social inclusion may decrease mental and physical health [16], highlighting the need for holistic, multidisciplinary LTFU and adequate care structures.

The LE‐Na trial (Evaluation and implementation of multidisciplinary, standardized, guideline‐based long‐term follow‐up care for adult survivors of childhood cancer in Germany) was designed to address this gap in healthcare provision with the aim of establishing, in the long term, a German Childhood Cancer Survivor (GCCS) cohort to obtain further information on late effects and LTFU. We now present interim results from the first recruitment months, focusing on the following two major objectives:

– To evaluate the recruitment strategies and acceptance of the nationwide LTFU care offer among adult CCS, identifying the most suitable approach for informing CCS about LTFU care.
– To assess the feasibility and potential of a structured guideline‐based psychosocial screening implementation in LTFU for CCS.

Based on this first assessment, the recruitment and psychosocial screening within the LE‐Na trial will be optimized to ensure efficient care pathways that meet CCS's needs.

## 2 Methods

### 2.1 Study Design and Setting

The prospective LE‐Na trial is conducted in 12 German specialized and multidisciplinary LTFU centers (Berlin, Bonn, Dresden, Erlangen, Frankfurt am Main, Freiburg, Hamburg, Lübeck, Mainz, Munich, Sankt Augustin, Tübingen) of which 11 contributed data to this analysis [17]. Within a five‐year study period (from 01/2023 until 12/2027), over 5000 CCS who have not yet received standardized LTFU are systematically invited for study participation, which includes regular LTFU care visits, adapted to their risk of developing late effects, as well as documentation of their medical and psychological data in a previously implemented database [18]. Considering the 10 centers initially participating in the LE‐Na consortium, it was estimated that centers would enroll an average of 100 patients annually over the study period.

### 2.2 Participants and Recruitment

Participants diagnosed with cancer before the age of 18 years and 5 years or more post‐treatment are eligible if they are 18 years or older at study inclusion, have German language proficiency, and can provide informed consent. Individuals receiving treatment (for a subsequent neoplasm) and survivors who previously attended a comparable LTFU care program are not eligible.

Recruitment comprises three strategies, with information on the study and LTFU offer (1) sent out via the GCCR, (2) provided at regular transition from pediatric to adult care, or (3) provided via media. For the latter, a study was established as a shared online platform providing information about all participating LTFU centers. In addition, efforts were made to promote the LE‐Na study through publications in relevant outlets, such as magazines for general practitioners. Furthermore, the study centers participated in informational events and conferences, where they presented the study and distributed flyers. During LTFU consultations, participating CCS are stratified according to their risk for late effects based on a previously published classification [19, 20]. CCS will be informed about the stratification results and on type and frequency of follow‐up examinations (including necessary diagnostic examinations such as electrocardiogram, echocardiography, thyroid or abdominal ultrasound, as well as bone mineral density assessment in accordance with current clinical guideline recommendations) during the study period based on their risk group (RG) using a stratification model described in a previously published publication [19, 20].

RG1 with a low risk for developing late effects comprises CCS who had only required surgical therapy (with exception of CCS with a central nervous system [CNS] tumor) as well as CCS of acute lymphatic leukemia (ALL) or non‐hereditary retinoblastoma who received chemotherapy only. CCS with medium risk for late effects (RG2) had received chemotherapy (excluding ALL and non‐hereditary retinoblastoma) or, in case of a CNS tumor, had merely undergone surgical therapy. RG3 includes CCS at high(er) risk for late effects, such as after hematopoietic stem cell transplantation and/or irradiation.

According to a priori estimations and experiences of study recruitment of CCS in Germany, we hypothesized that 50% of participants would be recruited via the GCCR, 40% via transition, and 10% via media presence.

### 2.3 Material

Medical data, including baseline information such as patient demographics (date of birth, sex, height, and weight), diagnosis and treatment details as well as longitudinal data (on endocrinological findings, cardiovascular disease, hearing, vision, substance use, vaccination status, appetite, mobility, neuropsychiatric disorders, and disorders of other organ systems; relapses and subsequent neoplasms) will be collected from hospital case files in the participating centers.

Findings are documented in a standardized manner to prospectively record the prevalence of late effects in a clinical follow‐up database (further details have been published [18]). As part of data collection for the primary study endpoints, participating CCS are requested to fill out study evaluation questionnaires on health‐related self‐efficacy as well as satisfaction with the provision of care.

Data from CCS diagnosed between 1980 and 1999 who were contacted by the GCCR provide the basis for a non‐responder analysis of current age, sex, oncological diagnosis, time of diagnosis as well as age at time of diagnosis of participants and current non‐participants.

### 2.4 Questionnaires

A comprehensive questionnaire battery using well‐established assessment tools, embedded into the standardized LTFU structure, covers relevant mental health issues experienced by CCS. These tools enable screening and offer a basis for obligatorily following counseling sessions. Assessment tools were chosen according to previous research stating CCS have an increased risk for affective disorders such as depression and anxiety, PTSD, general psychological distress, suicidal ideation, as well as fatigue [9, 21]. Updated German normative values are available for almost all of the questionnaires (e.g., [22]). This questionnaire battery follows three study evaluation questionnaires via a study tablet during the same session, completing the design of the study as described in [17].

#### 2.4.1 Psychological Distress, Depression, Anxiety

Patient Health Questionnaire‐4 (PHQ‐4 [23]) is an ultra‐brief screening tool with two items related to anxiety and two assessing depressive symptoms. It enables a rapid appraisal of general psychological distress and has been validated for use in the general population. A cumulative score of six or more indicates clinically relevant distress. A score of ≥ 3 in the first two items indicates relevant depressive symptoms, which triggers the assessment of the next seven items of the PHQ‐9 (see below).

Patient Health Questionnaire‐9 (PHQ‐9 [24]) is a nine‐item instrument assessing depressive symptoms' severity based on Diagnostic and Statistical Manual of Mental Disorders (DSM)‐IV criteria (low mood, anhedonia, fatigue, changes in appetite and sleep, difficulty concentrating, psychomotor changes, feelings of worthlessness, and suicidal ideation), widely used for both screening and monitoring of treatment response. If the short version of the screening (see below) is used, the PHQ‐9 assessment is not activated and counselors are asked to explore further depressive symptoms during consultation.

Distress‐Thermometer [25] is a single‐item tool to self‐report psychological distress over the past week on a visual analog scale from 0 to 10. In the long version of the screening, it is supplemented by a problem checklist covering emotional, physical, practical, family, and spiritual concerns. This measure enables a quick and broad identification of distress and related problems.

#### 2.4.2 Posttraumatic Stress and Trauma Symptoms

Impact of Event Scale‐Revised (IES‐R [26]) is a 22‐item instrument measuring PTSD symptoms following a traumatic event, including cancer illness or treatment. It captures three core domains of PTSD: intrusion, avoidance, and hyperarousal.

PTSD Screening Item (IGHG; [9]) is a single‐item screening tool to identify survivors of childhood, adolescent, and young adult cancers in need of further psychological evaluation regarding PTSD symptoms.

#### 2.4.3 Fatigue

EORTC (European Organization for Research and Treatment of Cancer) QLQ (Quality Of Life Questionnaire)—FA12 [27] is a 12‐item module assessing cancer‐related fatigue in clinical and research settings by evaluating physical, emotional, and cognitive fatigue, as well as the extent to which fatigue interferes with daily functioning. The scale is used internationally in conjunction with the EORTC QLQ‐C30 to provide a comprehensive picture of fatigue burden.

#### 2.4.4 Quality of Life

EORTC QLQ‐C30 [28] is a 30‐item questionnaire evaluating quality of life in cancer patients across multiple functional domains (physical, role, emotional, cognitive, and social), various symptoms (e.g., pain, fatigue, nausea), and global health status. In this study, items from the QLQ‐C30 are additionally used to inform study evaluation, and are thus not only implemented for screening reasons.

#### 2.4.5 Healthcare Utilization, Demographics, and Social Outcomes

Further, single‐item questions collected key sociodemographic variables (such as age, sex, educational and occupational outcomes, living situation and partnership status, parenthood and desire to have children, as well as needs for counseling on degree of disability and disability pension) and data on past or current use of psychosocial and psychiatric care. These data allow for subgroup analysis and contextual interpretation of patient‐reported outcomes, but may also serve to specify somatic risk factors for precarious social outcomes.

To account for survivors with reduced cognitive capacities, an alternative short version of the screening battery was compiled [17], consisting of EORTC QLQ C30, EORTC QLQ FA12, the Distress‐Thermometer without problem areas, PHQ‐4, and a PTSD screening question [9]. To compensate for information loss, it was aimed to increase exploration depth in the mandatory ensuing psychosocial consultations (Figure S1). Participants are free to choose between versions, but are encouraged to complete the long version of the screening battery after having filled out the study questionnaires [17]. On average, completion of the whole battery takes 25 min. Neither version allows for missing answers, but participants can decide to discontinue if completed on a tablet. In case the tablet is not preferred or available, paper backup versions are used, which may lead to missing data.

### 2.5 Statistical Analysis

All statistical analyses were conducted using IBM SPSS Statistics, Version 29. Descriptive analyses encompassed CCS, whose data were documented and completed in the database until March 31, 2025, aligning the scope and time‐point of interim analysis with study requirements. All continuous variables are presented as arithmetic means with standard deviations (SD) and as median values with interquartile ranges (IQR). Group comparisons by RG or recruitment groups and numerical variables were performed using the Kruskal–Wallis test to compare means of metric variables, such as age and time since diagnosis, and chi‐squared test for other non‐metric variables such as sex, RG, and oncological diagnoses. Reported *p* values are of purely descriptive nature; asymptotic *p* values were given where applicable. Oncological diagnoses were grouped into main categories for comparative analyses (see Table 1).

**TABLE 1:** Recruitment overview and general characteristics of our cohort (adult childhood cancer survivors enrolled into the LE‐Na study and database between 07/2023 and 03/2025 (*n* = 300)).

Characteristics	MD	Transition (*n* = 155)	GCCR (*n* = 121)	Media presence (*n* = 24)	Total (*n* = 300)	*p* *
Age at time of inclusion^a^ (years)	0				300	
Mean ± SD (range)		22.6 ± 5.8 (18.0–45.0)	39.3 ± 6.3 (23.1–57.0)	31.1 ± 7.8 (20.1–52.1)	30.0 ± 10.1 (18.0–57.0)	< 0.001
Median (IQR)		20.1 (3.9)	39.0 (9.0)	30.0 (10.5)	28.1 (18.0)	
Sex (*n*)	0				300	
Female		82 (52.9%)	71 (58.7%)	11 (45.8%)	164 (54.7%)	0.420
Male		73 (47.1%)	50 (41.3%)	13 (54.2%)	136 (45.3%)	
Risk group (*n*)	0				300	
1—Low risk of late effects		44 (28.4%)	33 (27.3%)	6 (25.0%)	83 (27.7%)	0.075
2—Intermediate risk of late effects		54 (34.8%)	28 (23.1%)	4 (16.7%)	86 (28.7%)	
3—High risk of late effects		57 (36.8%)	60 (49.6%)	14 (58.3%)	131 (43.7%)	
Oncological diagnosis (*n*)	0				300	
Leukemia		61 (39.4%)	58 (47.9%)	8 (33.3%)	127 (42.3%)	0.025
Lymphoma		38 (24.5%)	12 (9.9%)	7 (29.2%)	57 (19.0%)	
Central nervous system tumor		10 (6.5%)	10 (8.3%)	3 (12.5%)	23 (7.7%)	
Bone and soft‐tissue tumor		16 (10.3%)	18 (14.9%)	3 (12.5%)	37 (12.3%)	
Embryonal tumor^b^		23 (14.8%)	23 (19.0%)	3 (12.5%)	49 (16.3%)	
Others^c^		7 (4.5%)	0	0	7 (2.3%)	
Age at time of diagnosis (years)	0				300	
Mean ± SD (range)		8.5 ± 5.2 (0–17)	5.2 ± 4.0 (0–15)	8.1 ± 5.5 (0–16)	7.2 ± 5 (0–17)	< 0.001
Median (IQR)		9.0 (10.0)	5,0 (6.0)	8.0 (11.7)	6.1 (8.8)	
Time since diagnosis^d^ (years)/(*n*)	0				300	
Mean ± SD (range)		13.5 ± 7.1 (5.0–36.1)	33.5 ± 5.7 (8.0–45.1)	22.5 ± 8.2 (6.0–38.0)	22.3 ± 11.6 (5.0–45.1)	< 0.001
Median (IQR)		12.1 (10.0)	33.0 (8.0)	21.5 (11.5)	22.0 (19.7)	
5 years		8 (5.2%)	0	0	8 (2.7%)	
6–10 years		57 (36.8%)	1 (0.8%)	1 (4.2%)	59 (19.7%)	
11–15 years		37 (23.9%)	0	3 (12.5%)	40 (13.3%)	
> 15 years		53 (34.2%)	120 (99.2%)	20 (83.3%)	193 (64.3%)	
(First) Relapse	0				300	
Yes (*n*)		13 (8.4%)	9 (7.4%)	1 (4.2%)	23 (7.7%)	0.764

*Note:* Percentages may not add up to 100% due to rounding; percentages of the same absolute numbers might differ as only valid values were analyzed per variable.

Abbreviations: GCCR, GERMAN Childhood Cancer Registry; MD, missing data.

^a^ Referring to age at time of first clinical follow‐up examination.

^b^ Consisting of: neuroblastoma, nephroblastoma, retinoblastoma, yolk sac tumor, ganglious desmoplastic tumor, ganglioneuroma, seminoma, germ cell tumor with mixed histology, teratoma.

^c^ Others: extracranial germ cell tumor, neuroendocrine tumor, adrenal cortex tumor, colon cancer, Langerhans cell histiocytosis.

^d^ Time span between date of primary oncological diagnosis and first clinical follow‐up examination.

* *p* values are provided for descriptive purposes only. Differences in age at inclusion and time since diagnosis between the subgroups are reported for completeness, although expected and inherent to the respective recruitment strategies.

## 3 Results

### 3.1 Setup of Study Centers and Recruitment Overview

By the time of interim analyses, 11 centers had consecutively initiated patient recruitment, and one had ongoing regulatory challenges. On average, centers had recruited over a period of 11 months (Table S1) and a total of 300 CCS between 07/2023 and 01/2025 (first patient in to last date included for interim analyses).

### 3.2 Participant Characteristics

In the analyses, we included 300 CCS with fully completed medical data sets up until the cut‐off date, of which 121 (40.3%) were recruited through GCCR invitation, 155 (51.7%) through structured transition, and 24 (8%) through media presence (see Table 1). Nearly half (131/300; 43.7%) of the participants had a high risk for late effects (CCS recruited via the GCCR 49.6%; transition 36.8%; and media presence 58.3%). The distribution of sex, RG, and relapses did not differ substantially between recruitment groups. Age at time of inclusion, age at time of diagnosis, and time since diagnosis varied notably between recruitment groups, with the GCCR subgroup comprising the oldest participants and the longest time since diagnosis. The distribution of oncological diagnoses differed between recruitment groups, of which leukemia (42.3%), lymphoma (19.0%), and embryonal tumor (16.3%) were the most frequent diagnoses.

### 3.3 Non‐Responder Analysis, Carried Out by the GCCR

Out of the 1470 CCS contacted by the GCCR until the time‐point of the interim analysis, 282 (19%) had agreed to participate in the study. However, only 121 with completed data sets were considered for this interim analysis (see above). Those identified as (current) non‐responders (1121, 76%) had not yet responded by contacting the study center. The remaining 5% (67 CCS) were deceased, did not meet the inclusion criteria (any longer), actively declined participation, or mail was undeliverable (see Table 2).

**TABLE 2:** Non‐responder analysis, carried out by the German Childhood Cancer Registry (GCCR).

Characteristics	Participants (*n* = 282)	Current non‐participants (*n* = 1121)	*p* *
Age on 31.01.2025 (years)			
25–34	78 (28%)	326 (29%)	0.842
35–44	148 (52%)	567 (51%)	
45–58	56 (20%)	228 (20%)	
Sex			
Female	154 (55%)	464 (41%)	< 0.001
Male	128 (45%)	657 (59%)	
Oncological diagnosis			
Leukemia	133 (47%)	437 (39%)	0.050
Lymphoma	24 (9%)	150 (13%)	
CNS tumor	28 (10%)	136 (12%)	
Bone and soft‐tissue tumor	35 (12%)	114 (10%)	
Embryonal tumor^a^	51 (18%)	231 (21%)	
Others^b^	11 (4%)	53 (5%)	
Time of diagnosis			
1980–1989	103 (37%)	383 (34%)	0.457
1990–1999	179 (63%)	738 (66%)	
Age at time of diagnosis (years)			
< 1–4	152 (54%)	540 (48%)	0.085
5–14	130 (46%)	581 (52%)	

*Note:* Percentages were rounded to whole numbers.

Abbreviation: CNS, central nervous system.

^a^ Neuroblastoma, retinoblastoma, and nephroblastoma.

^b^ Hepatoblastoma, germ cell tumor, other malignant epithelial neoplasm, and other specified malignant tumor.

* *p* values (generated via chi‐squared tests online: https://www.icalcu.com/stat/chisqtest.html) are provided for descriptive reasons only.

Participants and current non‐participants did not differ considerably in age, time of diagnosis, or age at time of diagnosis. However, participants were more often female (55% vs. 41%, *p* < 0.001), and there was a slight difference in oncological diagnoses (*p* = 0.050), with participants having been diagnosed with leukemia more and lymphoma or embryonal tumors less frequently.

We further carried out a voluntary, anonymous online survey offered to all non‐responders in which CCS could indicate reasons for non‐attendance: 22 CCS responded in total, of whom five had no interest in the offer, eight found the distance to the nearest LTFU center too far, one was already being followed up in a LTFU center, five found LTFU care by their general practitioner to be sufficient, and three had other reasons.

### 3.4 Psychosocial Screening

Almost all participants (299/300, 99.7%) completed the screening for psychosocial distress and risk factors, with 265 (88.6%) survivors completing the long version and 34 (11.4%) completing the alternative short version (see Table 3). Participants who completed the long version of psychosocial questionnaires did not differ from those who completed the short version regarding age, sex, RG, or oncological diagnoses. A difference was seen in activation of PHQ‐9, respectively a PHQ‐4 score of ≥ 3 in the first two items of the PHQ‐4, with a considerably larger proportion of CCS in the short version affected.

**TABLE 3:** Overall study participation in the evaluation and psychosocial questionnaires and characteristics of adult childhood cancer survivors enrolled into the LE‐Na study and database between 07/2023 and 03/2025 (*n* = 300).

	MD	Total	Long version of psychosocial questionnaires^a^	Short version of psychosocial questionnaires^b^	*p* *
Completion of psychosocial questionnaires	1	299	265/299 (88.6%)	34/299 (11.4%)	
PHQ‐9^c^ activated/PHQ‐4^d^ score ≥ 3			40/265 (15.1%)	10/34 (29.4%)	< 0.001
Evaluation questionnaires^e^	2	298	264/265 (99.6%)	33/34 (97.1%)	
Age at time of inclusion^f^ (years)	0	300			
Mean ± SD (range)		30.0 ± 10.1 (18.0–57.0)	25.8 ± 9.5 (18.0–48.0)	30.5 ± 10.1 (18.0–57.0)	0.531
Median (IQR)		28.1 (18.0)	21.0 (17.3)	30.0 (8.5)	
Sex (*n*)	0	300			
Female		164 (54.7%)	19 (55.9%)	144 (54.3%)	0.864
Male		136 (45.3%)	15 (44.1%)	121 (45.7%)	
Risk group (*n*)	0	300			
1—Low risk of late effects		83 (27.7%)	11 (32.4%)	72 (27.2%)	0.763
2—Intermediate risk of late effects		86 (28.7%)	10 (29.4%)	76 (28.7%)	
3—High risk of late effects		131 (43.7%)	13 (38.2%)	117 (44.2%)	
Oncological diagnosis (*n*)	0	300			
Central nervous system tumor		23 (7.7%)	3 (8.8%)	19 (7.2%)	0.728
All other diagnoses^g^		277 (92.3%)	31 (91.2%)	246 (92.8%)	
Age at time of diagnosis (years)	0	300			
Mean ± SD (range)		7.2 ± 5 (0–17)	7.1 ± 4.6 (0–15)	7.2 ± 5.1 (0–17)	0.988
Median (IQR)		6.1 (8.8)	7.0 (4.0)	6.1 (9.0)	

*Note:* Percentages may not add up to 100% due to rounding; percentages of the same absolute numbers might differ as only valid values were analyzed per variable.

Abbreviation: MD, missing data.

^a^ Consisting of: EORTC QLQ C30 + EORTC QLQ FA12, Distress‐Thermometer, PHQ‐4, PHQ‐9 if PHQ‐4 first two items result ≥ 3, Impact of Event‐Scale (IES‐R), Questionnaire on Living Situation.

^b^ Consisting of: EORTC QLQ C30 + EORTC QLQ FA12, shortened Distress‐Thermometer (without problem areas), PHQ‐4, PTSD‐screening item, Questionnaire on Living Situation.

^c^ Last seven questions of PHQ‐9, only activated upon surpassing the threshold in PHQ‐4 first two items (≥ 3) in the long version of the psychosocial questionnaires.

^d^ Patient Health Questionnaire‐4 (≥ 3 referring to the sum of the first two items).

^e^ Consisting of: General Self‐Efficacy‐Scale (SWE), Patient Satisfaction Questionnaire‐Short Form (PSQ‐18) & Healthcare Self‐Efficacy‐Scale (HCSE).

^f^ Referring to age at time of first clinical follow‐up examination.

^g^ Consisting of: leukemia, lymphoma, bone and soft‐tissue tumor, embryonal tumor and others.

* *p* values provided for descriptive reasons only.

### 3.5 Psychosocial Distress, Measured by the Distress‐Thermometer

**FIGURE 1:**
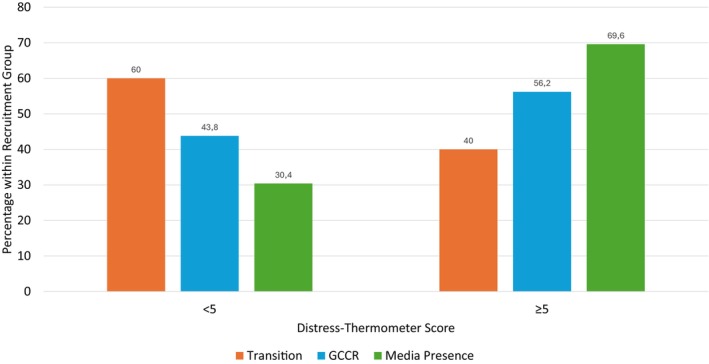
Cross‐table Distress‐Thermometer score to recruitment group. GCCR, German Childhood Cancer Registry.

Self‐reported scores on the Distress‐Thermometer (see Figure 1) ranged from 0 (no distress) to 10 (extreme distress). Among participants recruited via the GCCR or by media presence, most indicated upper distress scores of 5–10 (56.2% and 69.6%, respectively), while CCS recruited via transition peaked at lower scores, with 60% displaying scores of 0–4 (*p* = 0.003). Overall, half of all participants (146/299; 48.8%) indicated a score of ≥ 5 on the Distress‐Thermometer and thus, relevant psychosocial distress.

The distribution of CCS across the two distress categories was relatively balanced in all RG: A slightly higher proportion of RG1 reported clinically relevant distress (score ≥ 5; 53.1%), whereas RG2 and RG3 were more frequently classified in the lower distress category (score < 5; 54.7% and 51.5%, respectively).

### 3.6 Sociodemographics

The sociodemographic characteristics of the cohort revealed a relatively balanced sex distribution across all three RGs (see Table 4), with a slight predominance of female participants (54.7%). More than half of the participants reported being in a solid partnership (57.2%), while 41.5% identified as single, with minor differences between RG. Most participants (83.6%) were living with others (i.e., a partner and/or children or in a shared flat), and only a small proportion reported living alone (14.0%) or in assisted or other housing arrangements. Educational status varied across RGs, with 26.1% in total reaching ISCED (International Standard Classification of Education) level 6 or 7 (Bachelor's or Master's or equivalent level). Among participants who indicated to be employed, 67.6% were working full‐time, 26.9% part‐time, and only 5.5% were marginally employed.

**TABLE 4:** Sociodemographic characteristics of our cohort.

Sociodemographics	MD	Total	RG1 (*n* = 83)	RG2 (*n* = 86)	RG3 (*n* = 131)	*p* *
Sex	0	300				
Female		164 (54.7%)	46 (55.4%)	43 (50.0%)	75 (57.3%)	0.569
Male		136 (45.3%)	37 (44.6%)	43 (50.0%)	56 (42.7%)	
Partnership status	1	299				
Single^a^		124 (41.5%)	29 (34.9%)	36 (41.9%)	59 (45.4%)	0.329
Changing partnerships		4 (1.3%)	1 (1.2%)	0	3 (2.3%)	
In solid partnership^b^		171 (57.2%)	53 (63.9%)	50 (58.1%)	68 (52.3%)	
Living situation	1	299				
With others^c^		250 (83.6%)	70 (84.3%)	73 (84.9%)	107 (82.3%)	0.0936
Alone		42 (14.0%)	12 (14.5%)	10 (11.6%)	20 (15.4%)	
In an assisted living facility		2 (0.7%)	0	1 (1.2%)	1 (0.8%)	
Other living situation		5 (1.7%)	1 (1.2%)	2 (2.3%)	2 (1.5%)	
Highest level of education	1	299				
ISCED 1^d^		1 (0.3%)	0	0	1 (0.8%)	0.085
ISCED 2^e^		59 (19.7%)	21 (25.3%)	22 (25.6%)	16 (12.3%)	
ISCED 3^f^		146 (48.8%)	34 (41.0%)	45 (52.3%)	67 (51.5%)	
ISCED 5^g^		15 (5.0%)	4 (4.8%)	5 (5.8%)	6 (4.6%)	
ISCED 6 or 7^h^		78 (26.1%)	24 (28.9%)	14 (16.3%)	40 (30.8%)	
Worktime, hours per week	118	182				
Marginal employment (≤ 10 h)		10 (5.5%)	2 (4.2%)	3 (6.5%)	5 (5.7%)	0.459
Part‐time (11–34 h)		49 (26.9%)	11 (22.9%)	9 (19.6%)	29 (33.0%)	
Full‐time (≥ 35 h)		123 (67.6%)	35 (72.9%)	34 (73.9%)	54 (61.4%)	

Abbreviation: MD, missing data.

^a^ Consisting of: single, divorcing and single, divorced and single.

^b^ Consisting of: married, in solid relationship, divorcing and in new relationship, divorced and in new relationship, divorced and remarried.

^c^ Consisting of: with parents, with a partner, with a partner and children, with children, in a shared flat; ISCED = International Standard Classification of Education.

^d^ Consisting of: no vocational qualification and school discontinued with no school leaving certificate.

^e^ Consisting of: no vocational qualification and ISCED 2 in school‐leaving certificate, currently in training and ISCED 2 in school‐leaving certificate, currently in school and aiming at a school‐leaving certificate.

^f^ Consisting of: no vocational qualification and ISCED 3 in school‐leaving certificate, currently studying and ISCED 3 in school‐leaving certificate, university studies or training discontinued without degree and ISCED 3 in school‐leaving certificate, completed vocational training, completed vocational training at a vocational/commercial school.

^g^ Master craftsman qualification.

^h^ Consisting of: university degree (Bachelor's or Master's), applied sciences degree.

* *p* values are of purely descriptive nature.

## 4 Discussion

Following cancer treatment, only an estimated 5% of the growing number of adult CCS are receiving specialized LTFU care in Germany. There is still a lack of comprehensive nationwide care structures that address both the somatic and the diverse psychosocial late effects, as well as informational needs in CCS. Qualitative studies emphasize the necessity of care and information tailored to CCS's late‐effects risk, psychosocial needs, and personal preferences by providing integrated psychosocial support, easily accessible centralized and specialized care, and individualized follow‐up services [29]. The LE‐Na study closes this gap by systematically inviting CCS who have not yet received LTFU care and implementing a standardized and evidence‐based care structure.

In this interim analysis, we evaluated different recruitment strategies to inform CCS about the emerging multidisciplinary services and the integration of standardized psychosocial screening. Recruitment via media presence, although the smallest group in our collective, yielded the highest proportion of high‐risk survivors to present at the LTFU clinics, suggesting the potential of targeted outreach for vulnerable subgroups. The structured transition pathway proved particularly effective in reaching survivors early and ensuring engagement with LTFU services. As almost half of the participants indicated relevant psychosocial distress, illustrated, for example, by high scores on the Distress Thermometer, the need for integrated psychosocial services as part of recommended routine LTFU [30, 31] is further underscored. The results provide a strong basis for continued implementation and expansion of multidisciplinary LTFU networks for CCS in Germany.

The LE‐Na study initially included 10 centers, already providing a wide network throughout Germany. During the course of time, further centers were involved, particularly in underserved areas, resulting in a growing number of centers offering standardized care [17]. Centers commenced with recruitment consecutively and recruited a growing number of patients per quarter over time. This allows the necessary structural adaptations for providing qualitative care in an already burdened health care system with financial strains and reduced personal capacities. Delays in study site initiation were frequently associated with administrative factors. During regular meetings involving all participating sites in the LE‐Na study, these barriers were systematically identified and addressed to facilitate the initiation of additional sites, serving as a blueprint for future initiatives aiming at improving accessibility.

Recruitment of CCS comprised a broad strategy. The fact that a high proportion of CCS (almost half of the cohort) with a high risk for late effects across all recruitment paths contacted the centers is an important achievement, as this especially vulnerable group potentially benefits most from specialized LTFU care, despite posing a potential selection bias for the interim and future analyses. It is plausible that health‐conscious individuals or CCS burdened by late effects may have been more motivated to participate in the LE‐Na study [32], potentially limiting the generalizability of our findings to the broader CCS population, yet it gives valuable insight into how CCS who have not yet participated in LTFU can be motivated. In this context, the role of media presence should be more strategically integrated into communication strategies, not only within the scope of research, but also as part of the broader clinical care concept. However, further awareness also in low‐ and medium‐risk groups needs to be warranted by appropriate and early counseling. Recruitment via transition ensures seamless care from oncological aftercare to LTFU. The CCS included in our study were not receiving mandatory LTFU care at the time of enrollment. Adherence to LTFU remains a challenge: in a French cohort, only 33% of adult CCS attended at least one LTFU visit over an 8‐year period [33].

While cancer registries are important partners in the recruitment of CCS for study participation, only 19% of those contacted for the LE‐Na Study and associated LTFU care offer had presented in one of the specialized centers at the time of data analysis. Non‐responder analyses among those contacted by the GCCR revealed that male CCS were slightly underrepresented in the participant sample, indicating a well‐known potential sex bias in recruitment and participation [34, 35]. As defined in the recruitment strategy, those who presented via the GCCR were the oldest at the time of enrollment, with the longest time since treatment. Given that treatment intensity has changed over the past decades, and awareness of late effects has specifically increased over the past two decades, reaching out to these very long‐term survivors (e.g., treated in the 80s, 90s) is of paramount importance, as by now many will already have manifested late effects, which require multidisciplinary care.

Given the increased prevalence of mental health in CCS, the standardized implementation of a psychosocial screening is therefore a central aspect of the LTFU care. Guideline recommendations range from a large set of validated screening tools. Within the LE‐Na study, we offered a long and short version of the psychosocial screening to acknowledge potential burden on CCS. Most participants completed the long version, supporting the feasibility of such in a structured clinical setting with a low‐threshold digital option. While originally designed to account for potential neurocognitive impairment as a result of cancer and therapy, in our cohort, those with answers indicating mental health issues were more likely to have used the short version. A relevant future adaption of the psychosocial screening may therefore include the integration of PHQ9 also in the short version. The majority of participants who had previously suffered a CNS tumor (and are thus at higher risk for neurocognitive impairment) completed the long version. This unexpected finding may be explained by a general selection bias. Those experiencing less severe neurocognitive sequelae were potentially more likely to participate, corresponding with known confounders for participation in studies (e.g., higher education).

One of the components of the screening, the Distress Thermometer, is an important tool during but also after cancer treatment to identify those in need of further psychological support, supporting the integration of tailored psychosocial interventions following screening [36, 37]. It is easy to use, and large comparison groups are available [38]. In our interim analyses, those who presented via media presence more often, as well as older CCS, reported higher levels of distress. This further supports the role of integration of varying recruitment strategies to address specific vulnerable groups and reach those with high psychosocial burden. When significant psychosocial distress was detected and/or the need for further psychosocial counseling or clarification was expressed, psychosocial workers could schedule another appointment, arrange a consultation, refer patients to support services or outpatient diagnostics, provide informational material or make alternative arrangements. The literature also notes that the Distress‐Thermometer should not be used as a stand‐alone tool, and that follow‐up strategies should be individualized based on CCS's specific psychosocial profile and preferences [39].

Besides somatic and mental health impairments, which will be fully analyzed at the conclusion of the LE‐Na study, the impact of cancer on personal development, adapting life goals and social relationships is well known [14]. The LE‐Na study, therefore, complements medical data and the psychological screening with social information to ensure a nuanced understanding of the individual's situation and a holistic approach in patient care to support a self‐determined life after cancer.

Ultimately, this study aims to improve future provision of LTFU care by adapting to CCS's needs. Despite the limitations related to cohort size and potential selection bias, the high quality of clinically validated, prospectively collected data represents a strength of the LE‐Na study. However, in the future, structured follow‐up visits and updates in comprehensive data collection and documentation will enhance the quality and reliability of our findings. Through the longitudinal design of the study, future analyses will support a better understanding of the trajectories of (psychosocial) health and late effects over time. Accordingly, this cohort serves as an important basis for future longitudinal analyses (German Childhood Cancer Survivor Study) and may complement larger international datasets to improve evidence‐based LTFU care strategies [40].

## 5 Conclusion

The LE‐Na study has implemented standardized LTFU care for CCS in a growing number of specialized centers throughout Germany since 2023 combined with systematic collection of prospective, clinically validated medical and psychosocial data. This interim analysis provides important insights into the implementation process by addressing recruitment and information pathways and evaluating the feasibility of an integrated, standardized psychosocial screening. Our results support the necessity of a combined recruitment/information strategy to reach out to specific, vulnerable groups (e.g., elderly, CCS at risk for mental health issues) contributing to closing the education gap. Integration of a comprehensive psychosocial screening is feasible and confirms the increased prevalence of psychosocial burden in the cohort of CCS. The ongoing national implementation and expansion of this database as part of the LE‐Na project, with consecutive participation of additional hospitals in the coming years, will yield clinically validated and regularly updated health and psychosocial data from German CCS following the model of a lifetime cohort study such as the St. Jude Lifetime Cohort Study (SJLIFE) [41].

## Author Contributions

**Martina B. Schaefer:** writing – review and editing. **Alexander Puzik:** writing – review and editing. **Magdalena Balcerek:** conceptualization, formal analysis, validation, visualization, writing – review and editing, writing – original draft, methodology. **Gabriele Calaminus:** conceptualization, writing – review and editing. **Madelaine Sleimann:** conceptualization, data curation, formal analysis, validation, visualization, writing – review and editing, writing – original draft, investigation, methodology. **Sebastian Michaelis:** writing – review and editing. **Rainer John:** writing – review and editing. **Sonja Schuster:** writing – review and editing. **Teresa Halbsguth:** writing – review and editing. **Judith Lohse:** writing – review and editing. **Inke König:** conceptualization, data curation, formal analysis, writing – review and editing. **Anke Neumann:** writing – review and editing, software, conceptualization, data curation, resources. **Melanie Kaiser:** formal analysis, writing – review and editing. **Sophia Hömberg:** writing – review and editing. **Irene von Luettichau:** writing – review and editing. **Jörg Faber:** writing – review and editing. **Thorsten Langer:** writing – review and editing, conceptualization, funding acquisition, supervision. **Desiree Grabow:** writing – review and editing, conceptualization. **Ann‐Kristin Kock‐Schoppenhauer:** conceptualization, software, data curation, writing – review and editing, resources. **Judith Gebauer:** writing – review and editing, writing – original draft, funding acquisition, visualization, formal analysis, conceptualization, methodology, supervision. **Franziska Wolters:** writing – review and editing. **Celina Arendt:** project administration, data curation, writing – review and editing. **Katja Baust:** conceptualization, formal analysis, validation, visualization, writing – original draft, writing – review and editing, methodology.

## Funding

This work was supported by H.W. & J. Hector Stiftung (project M 2117).

## Ethics Statement

This study was approved by the ethics committee of the University of Luebeck (registration number 2022–647).

## Consent

Complete written informed consent was obtained from all patients participating in the study.

## Conflicts of Interest

F.W. received travel grants from GlaxoSmithKline and Alexion. The other authors declare no conflicts of interest.

## Supporting information


**Figure S1:** Enrollment of childhood cancer survivors and consultation process of standardized long‐term follow‐up care within the LE‐Na trial.
**Table S1:** Recruitment overview.

## Data Availability

The data that support the findings of this study are available from the corresponding author upon reasonable request.
