# Supplementary material for: Feasibility of Integrating a Standardized Psychosocial Follow‐Up After Childhood Cancer—An Interim Analysis of the LE‐Na Study
Source: Cancer Med. 2026 Aug 4;15(8):e72148. doi: 10.1002/cam4.72148 (PMC13435696; doi:10.1002/cam4.72148)
Supplement: Supplementary file 1 [file CAM4-15-e72148-s001.docx]

**Figure S1: Enrollment of childhood cancer survivors and consultation process of standardized long-term follow-up care within the LE-Na trial.**

**Table S1: Recruitment overview**

|  | **2023** | | | | | | **2024** | | | | | | | | | | | | **2025** | **Recruiting period (months)** |
| --- | --- | --- | --- | --- | --- | --- | --- | --- | --- | --- | --- | --- | --- | --- | --- | --- | --- | --- | --- | --- |
|  | 7 | 8 | 9 | 10 | 11 | 12 | 1 | 2 | 3 | 4 | 5 | 6 | 7 | 8 | 9 | 10 | 11 | 12 | 1 |  |
| Center 1 |  |  |  |  |  |  |  |  |  |  |  |  |  |  |  |  |  |  |  | 19 |
| Center 2 |  |  |  |  |  |  |  |  |  |  |  |  |  |  |  |  |  |  |  | 15 |
| Center 3 |  |  |  |  |  |  |  |  |  |  |  |  |  |  |  |  |  |  |  | 15 |
| Center 4 |  |  |  |  |  |  |  |  |  |  |  |  |  |  |  |  |  |  |  | 13 |
| Center 5 |  |  |  |  |  |  |  |  |  |  |  |  |  |  |  |  |  |  |  | 12 |
| Center 6 |  |  |  |  |  |  |  |  |  |  |  |  |  |  |  |  |  |  |  | 12 |
| Center 7 |  |  |  |  |  |  |  |  |  |  |  |  |  |  |  |  |  |  |  | 12 |
| Center 8 |  |  |  |  |  |  |  |  |  |  |  |  |  |  |  |  |  |  |  | 11 |
| Center 9 |  |  |  |  |  |  |  |  |  |  |  |  |  |  |  |  |  |  |  | 9 |
| Center 10 |  |  |  |  |  |  |  |  |  |  |  |  |  |  |  |  |  |  |  | 6 |
| Center 11 |  |  |  |  |  |  |  |  |  |  |  |  |  |  |  |  |  |  |  | 3 |
| **Average recruiting months** |  | | | | | |  | | | | | | | | | | | |  | **11,5** |
| **CCS included per year** | n = 19 | | | | | | n = 251 | | | | | | | | | | | | n = 30 | **total n=300** |

*highlighted in gray: active recruiting period per center
